# Harmony Transcatheter Pulmonary Valve Successfully Implanted via Transjugular Approach

**DOI:** 10.1016/j.jscai.2023.101207

**Published:** 2023-10-21

**Authors:** Pradyumna Agasthi, Allison K. Cabalka, Frank Cetta, Jason H. Anderson

**Affiliations:** a Department of Cardiovascular Medicine, Division of Structural Heart Diseases, Mayo Clinic, Rochester, Minnesota; b Department of Pediatric and Adolescent Medicine, Division of Pediatric Cardiology, Mayo Clinic, Rochester, Minnesota

**Keywords:** alternate vascular access, Harmony valve, pulmonary valve replacement, transjugular access

The Harmony transcatheter pulmonary valve (TPV) (Medtronic) was developed to address the extensive variability of right ventricular outflow tract (RVOT) anatomy in patients with significant pulmonary valve regurgitation. The valve has demonstrated excellent outcome data at 5 years.^1^ In patients with prior palliative surgery, there may be access site limitations to proceeding with TPV therapy. Herein, we present a 24-year-old nonverbal woman with history of Tetralogy of Fallot status postsurgical palliation including transannular RVOT reconstruction with a monocusp Gore-Tex patch. She was cachexic (body mass index 18 kg/m^2^, weight 37 kg) with exertional dyspnea and severe pulmonary valve regurgitation with moderate right ventricular enlargement and dysfunction. She was declined for surgical intervention due to high surgical risk and referred for TPV replacement. Computed tomography demonstrated favorable anatomy proximal to the pulmonary artery (PA) bifurcation with a prominent septal fold (Figure 1A).

**Figure 1:**
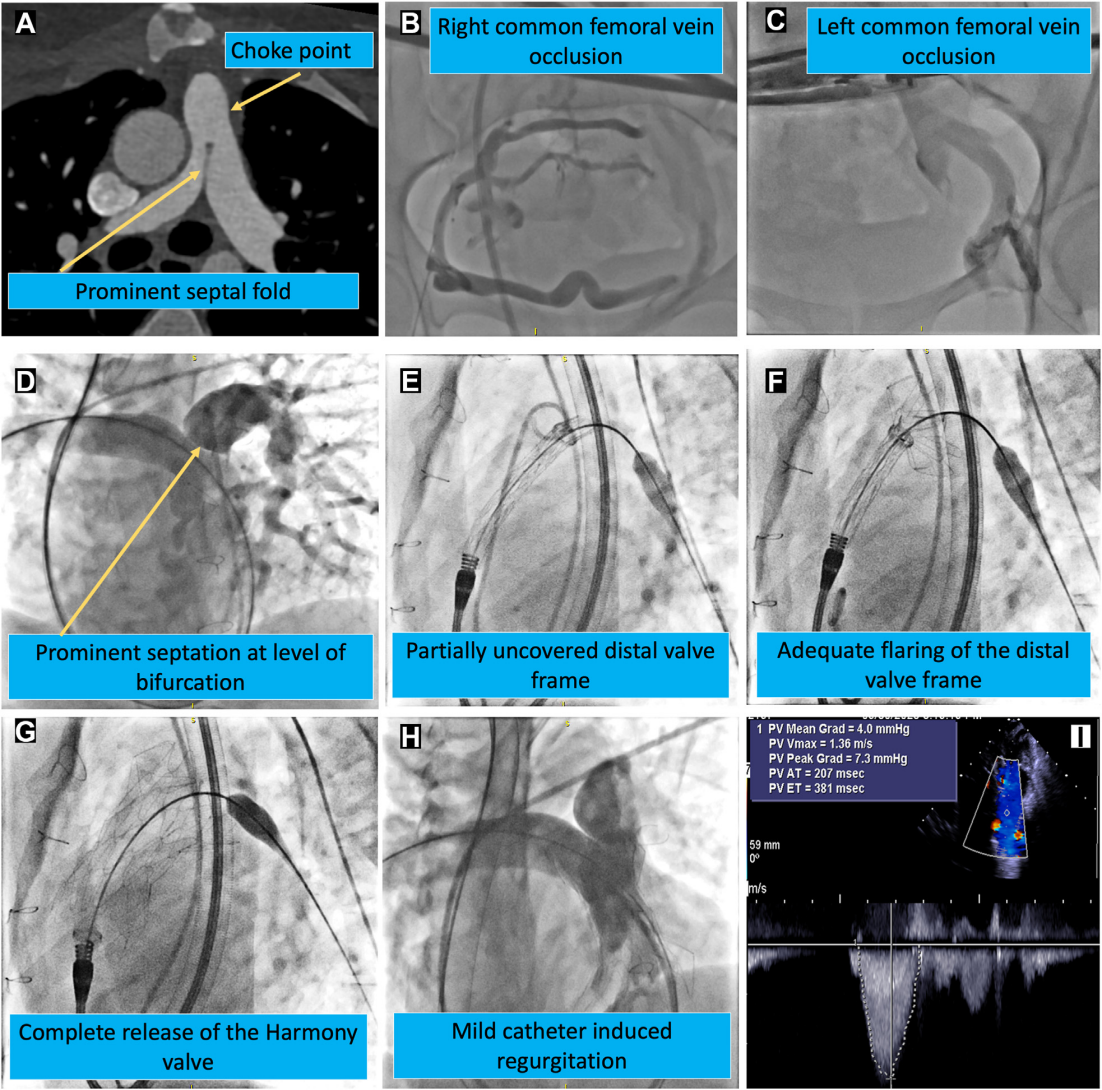
(**A**) Computed tomography demonstrates prominent septal fold proximal to the pulmonary artery bifurcation. (**B,C**) Bilateral common femoral vein occlusion. (**D-G**) Deployment of Harmony valve under angiographic guidance. (**H**) Repeat angiogram demonstrates mild catheter-induced regurgitation. (**I**) Intracardiac echocardiography demonstrates normal leaflet mobility with no regurgitation.

The patient had bilateral femoral venous occlusion (Figure 1B, C). The valve was therefore implanted from a right internal jugular approach with angiography performed via a secondary access from the left subclavian vein (Figure 1D, Supplemental Video 1). A 26F × 35-cm Gore DrySeal sheath (W.L. Gore & Associates) was positioned in the right atrium from the internal jugular vein. It was long enough to allow advancement to the RVOT if needed for valve retrieval, but the right atrial position was utilized to reduce impingement on the delivery system during advancement through the right ventricle. A 25-mm Harmony TPV was advanced into the right PA over a 1-cm soft tip Amplatz SuperStiff exchange wire (Abbott). Utilizing intermittent angiography, the valve was deployed via a right PA delivery technique. This involved gradual retraction of the partially deployed valve with antegrade force applied once the frame opened into the proximal left PA (Figure 1D-G). Repeat angiography demonstrated a competent valve with mild catheter-induced regurgitation (Figure 1H). Intracardiac echocardiogram demonstrated no prosthetic or periprosthetic regurgitation with mean systolic gradient of 4 mm Hg.

This case outlines the feasibility of performing transjugular TPV replacement with a Harmony valve in a patient with no femoral venous access. It represents a potential therapeutic opportunity for patients with congenital heart disease that may be suboptimal candidates for other therapy. The system is quite rigid, and deployment is very challenging from the internal jugular approach. We had initially assumed this deployment may be favorable to a femoral approach; however, unique to the Harmony valve as opposed to other pulmonary valve implants is the role of a delivery coil for a push/pull replacement technique. The RVOT bend in the system due to the internal jugular approach results in significant tension during deployment. Furthermore, the tip of the delivery catheter would not advance initially due to this tension, and thus, the system was flushed, which freed the end of the sheath to allow advancement. The utilization of a SuperStiff wire from the internal jugular approach also allowed the system to flex with greater ease during passage into the RVOT and is likely a favorable maneuver for small individuals (<20 kg). Overall, this case highlights the potential for Harmony TPV replacement from an internal jugular approach when necessitated, but it should be approached with an understanding that replacement may be more difficult.

## Supplementary material

Supplemental Video 1 Deployment of a Harmony TPV 25 transcatheter pulmonary valve from an internal jugular approach under fluoroscopic guidance.
